# Patient-Oriented Research Competencies in Health (PORCH) for patients, healthcare providers, decision-makers and researchers: protocol of a scoping review

**DOI:** 10.1186/s13643-018-0762-1

**Published:** 2018-07-19

**Authors:** Anastasia A. Mallidou, Noreen Frisch, Mary M. Doyle-Waters, Martha L. P. MacLeod, John Ward, Pat Atherton

**Affiliations:** 1 School of Nursing, University of Victoria, 3800 Finnerty (Ring) Road, Victoria, British Columbia V8P 5C2 Canada; 2 Centre for Clinical Epidemiology & Evaluation, Research Pavilion, 708A-828 West 10th Avenue, Vancouver, British Columbia V5Z 1M9 Canada; 3 Northern Health – UNBC Knowledge Mobilization Research Chair, University of Northern British Columbia, 3333 University Way, Prince George, BC V2N 4Z9 Canada; 4 Research Navigation and Project Lead, BC SUPPORT Unit, Suite 420 - 1367 West Broadway, Vancouver, BC V6H 4A7 Canada; 5 Training and Virtual Networking Platform, BC SUPPORT Unit, Suite 420 - 1367 West Broadway, Vancouver, BC V6H 4A7 Canada

**Keywords:** Patient-oriented research, POR competencies (knowledge, skills, attitudes), Scoping review, Patient and public involvement, Patient engagement

## Abstract

**Background:**

Patient-Oriented Research (POR) is a Canadian initiative for health research that refers to research processes informed by full and active patient involvement in all aspects of the research. Ideally, POR results in a wide dissemination of the research findings and the uptake of such findings in both clinical practice and health policy. The Canadian Institute for Health Research (CIHR) identifies four stakeholder groups that are involved in POR who are envisioned to take on a collaborative role in enacting this approach to research. Those stakeholder groups are patients, researchers, health care providers and healthcare decision-makers. To achieve collaboration among stakeholders in POR, tools, resources, education/training and capacity building are required for each stakeholder group engaged in this work. Therefore, this review focuses on understanding and articulating competencies needed by participants to engage in POR. The aim is to summarize existing knowledge on discrete POR competencies for the four stakeholder groups; to support collaboration among them for uptake and strengthening of POR; and to inform policy, education and future research. Accordingly, our research question is ‘What are the POR core competencies needed by patients, researchers, healthcare providers, and decision-makers?’ The main objectives are to (1) systematically explore the academic and grey literature on competencies needed for these stakeholder groups to engage in POR; (2) map the eligible publications and research gaps in this area; (3) gain knowledge to support collaboration among stakeholders; and (4) provide recommendations for further research to use competencies that emerge in developing stakeholder groups’ readiness to conduct POR.

**Methods/design:**

We will use a methodologically rigorous scoping review approach including formulation of the research question and development of the protocol; screening and identification of the literature; selection of relevant studies; data extraction; and collation, summary and report of the results. Our eligibility criteria include elements of population (patients, researchers, healthcare providers and decision-makers); concept (competencies: knowledge, skills, attitudes; and POR); context (level of involvement in research, settings, funding sources); study design (sample, stakeholder group, methodology, grey literature, theoretical framework); outcomes (primary: relevant to decision-making/policy and practice; and secondary: relevant to education and research); language (English, French); and timing (1990–2017). Registration with PROSPERO is not eligible for scoping reviews; so, it has not been registered.

**Discussion:**

Research on core competencies required to enact POR is in its infancy. In this review, we can articulate what is known and thought about competencies (knowledge, skills and attitudes) needed by individuals on POR research teams and ultimately provide knowledge that could impact research, practice, education and policy. Identification of competencies can contribute to design of healthcare professionals’ basic and ongoing educational programmes, patient training in research, and professional development activities for health care providers and decision-makers. In addition, knowledge of core competencies can permit individuals to evaluate their own readiness to enter POR research teams.

**Electronic supplementary material:**

The online version of this article (10.1186/s13643-018-0762-1) contains supplementary material, which is available to authorized users.

## Introduction/background

In the last two decades, research involving patients as partners has focused on clinical research and was defined (from the researcher perspective) as a revived science that has been the ‘Achilles’ heel of a disease’ [1]. There appeared to be a widespread agreement on the need to pursue a patient-oriented approach to clinical research [2–4], and more recently the Patient-Oriented Research (POR) Canadian initiative has been defined as research that is focused on the patient in the context of the whole person [5]. The unifying theme in POR has been the transformation of the individual researcher work experience towards a shared destination among ‘physicians and scientists from both academia and industry’ [5]. The scientists who involved patients in their work usually shook hands with the participating patients in the research, ‘the handshake test’ [6], and share four Ps: they have a *passion*ate curiosity about disease, are deeply involved with *patients*, have infinitive *patience* and endure grant *poverty* [6]. POR goes beyond these four Ps and shares many of the values of other global initiatives in changing the ways in which health research is conducted.

Recently, the Patient-Centred Outcomes Research Institute (PCORI; https://www.pcori.org) in the USA provided new insights into patient engagement in research. In the introductory section of its engagement rubric, PCORI states that ‘The evidence base for stakeholder engagement in clinical research is growing; it shows that engagement is associated with increased recruitment and retention of study populations; more patient-centred and culturally appropriate methods; and greater relevance of research questions and outcome measures.’ [7]. POR itself is a paradigm shift in the research process, due to its suggestion that research teams be comprised of patients, researchers, health care providers and decision-makers. In POR, patients play a fundamental and equal role as partners in the processes of determining research priorities, developing research questions, deciding on methodology and participating in data collection, analysis and dissemination of findings. In POR, patients are at the centre of the health system transforming practitioner-patient relationships from a paternalistic and provider-centred model to a patient-centred model characterized by patient autonomy. POR is similar to, but distinct from, INVOLVE, PPI and PCORI. INVOLVE was established in 1996 and funded by the National Institute for Health Research in the UK to support active public involvement in the National Health System (NHS), public health and social care research [8]. According to the current literature, PPI in research is conceptualized as, ‘doing research “with” or “by” the public, rather than “to”, “about” or “for” the public’ [9].

PCORI has as its basis the evaluation of health outcomes. As a result, there are many terms currently being used to describe research or research processes that have patient involvement (even to a limited degree), for example ‘patient-centred outcomes research’. While a specific definition of patient-centred outcomes research is lacking, it generally refers to research processes informed or endorsed by patients and does not include patients as partners for the entire scope of the research project. While elements of patient-centred outcome research may have influenced those working in POR, the two are philosophically distinct. POR includes patients in the entire research process, alongside researchers, healthcare providers and decision-makers, where each stakeholder group participates fully in each step of the process. This difference is also true of similar terms in use including patient-centred research, patient-centric research, person-focused research, community-based participatory research and so on. Thus, this review will include insights gained from all initiatives focusing on patients as research partners, but our team will be placing these insights within the POR framework that calls for a more inclusive research team and rigorous attention to the dissemination and use of research findings.

### CIHR SPOR and Patient Engagement Framework

In 2014, the Canadian Institutes of Health Research (CIHR) called patient representatives and patient engagement experts to participate in the Strategy for Patient-Oriented Research (SPOR) Patient Engagement Consultation Workshop (on January 9th) for developing the Patient Engagement (PE) Framework [10]. In this framework, POR is defined as ‘a continuum of research that engages patients as partners, focusses on patient-identified priorities and improves patient outcomes. This research, conducted by multidisciplinary teams in partnership with relevant stakeholders, aims to apply the knowledge generated to improve healthcare systems and practices’; while PE was defined as ‘Meaningful and active collaboration in governance, priority setting, conducting research and knowledge translation. Depending on the context, patient-oriented research may also engage people who bring the collective voice of specific, affected communities’ [10]. The PE Framework is designed to outline key opportunities for action; to establish key concepts, principles and areas for patient engagement to be adopted; and to set the stage for worthwhile collaborations in the identification of health research priorities and in the design and conduct of research projects. POR is ultimately aimed at achieving benefits that matter to patients such as improved health and access to the healthcare system, the right treatment at the right time, being an active and informed partner in healthcare, and quality of life that is tied to patient-oriented outcomes [10]. SPOR partners are ‘key stakeholders collaborating in POR such as the SUPPORT Unit jurisdictional leads for each province and territory, patients, researchers, policy makers, decision-makers, health organizations, provincial/territorial health authorities, academic institutions, charities and the pharmaceutical sector.’ [10]. Overall, POR can be summarized with the slogans ‘nothing about me without me’ and ‘one size does not fit all’ [11]. Additionally, POR can be understood as a new way for patients, health care providers, and health decision-makers to engage with researchers and research teams for a comprehensive understanding of patient needs and research priorities, and needs to adapt practice environments to take up research findings.

### POR competencies

To achieve active collaboration among patients, researchers, healthcare providers and decision-makers for POR, where all stakeholders are involved in all facets of the project, all stakeholders need to communicate meaningfully and efficiently. For this reason, tools, resources, education, training and capacity building are required to implement research collaborations among stakeholders. To enhance stakeholders’ measurable abilities and to achieve sustainable results in POR, each individual stakeholder group needs and competencies should be discussed and described. Learning and co-learning (e.g. researchers help patients and other stakeholders to understand the research process) are key principles in the patient-centred outcomes research [7]. We are unaware of any reviews that address patient-oriented research competencies for the four stakeholder groups we are focusing on in this study: patients, researchers, healthcare providers and decision-makers (as defined in Additional file 1). Considering that many governments require or expect the inclusion of patients as collaborators in research and the CIHR SPOR initiatives [10], this review is timely and will ultimately lead to the articulation of POR competencies that can be used to promote successful collaboration between the different stakeholder groups. Additionally, the importance of our work includes the following benefits for all partners in research:

- Better health research by sharing experiential knowledge and insights of patients with each of the other stakeholder groups.
- Acknowledgement of patient right to be involved in public-funded research [12].
- Improvement of the quality and relevance of care with patient involvement [13].

### Purpose and objectives

The purpose of the proposed scoping review is to summarize existing knowledge on POR competencies for four discrete stakeholders/collaborators: patients, healthcare providers, decision-makers, and researchers; to support collaboration among those stakeholders for the uptake and strengthening of POR; and to inform policy and further research. Our research question is ‘What are the core POR competencies of patients, researchers, healthcare providers, and decision-makers?’ Particularly, our main objectives are to:

- Systematically explore the extent of relevant theoretical and empirical literature as well as the grey literature (e.g. range, focus, nature of sources, volume) on POR competencies for the four stakeholder groups.
- Map the publications by identifying definitions (e.g. key themes) of stakeholders’ POR competencies and research gaps in this area.
- Gain knowledge to support further collaboration among stakeholders by articulating existing POR competencies and fostering the development of additional ones.
- Provide recommendations for further research to use competencies that emerge in developing stakeholder groups’ readiness to conduct POR and to use the competencies future research on topics such as evaluating the health outcomes of POR research.

## Methods

To address the purpose and objectives of the proposed scoping review, we will use the methodologically rigorous scoping review approach described by Arksey and O’Malley [14] and further developed by Levac and colleagues [15] and Colquhoun and associates [16]. This method includes five stages: (a) formulating the research question (and developing the protocol); (b) screening and identifying the literature (an iterative process); (c) selecting relevant studies; (d) extracting the data into charts; and (e) collating, summarizing and reporting the results.

### Formulating the research question and developing the protocol

The preliminary research question and objectives were presented in the previous section. This protocol, while a scoping (not systematic) review, was developed based on the ‘Preferred Reporting Items for Systematic Review and Meta-Analysis Protocols (PRISMA-P)’ [17–19] (see Additional file 2). PROSPERO (International Prospective Register of Systematic Reviews) only registers reviews that are focused on health related outcomes. Therefore, our protocol (a scoping, not systematic review) is not eligible for registration.

#### Inclusion/exclusion criteria

Scoping review questions tend to be broad, which is reflected in the inclusion criteria elements of population, concept and context [20] as well as study design, outcomes, language and timing.

##### Population

Four distinct stakeholder groups will be included in this review: patients, researchers, healthcare providers and decision-makers. *Patients*, defined by the CIHR’s Strategy for Patient-Oriented Research (SPOR), include people who have experience with a health condition, thereby including patients, caregivers, family and friends [10]. The INVOLVE [21] definition of patients includes ‘patients, potential patients, carers and people who use health and social care services as well as people from organisations that represent people who use services’. For this study, we agreed to use the CIHR SPOR definition augmented by our understanding that this definition can (and should) also include those populations or communities who may not have a ‘disease’ or a diagnosis or a ‘health condition’, but would be reasonably understood to comprise a population or group (i.e. those living in poverty, in a condition of homelessness, undergoing a life transition such as pregnancy and childbearing) that the POR approach is well suited to address. Our definition is not as broad as INVOLVE, but it does expand the literal meaning of the CIHR definition to provide opening for a more comprehensive and social understanding of ‘health conditions’ than from a strictly disease-oriented or diagnosis-oriented stance. The reasons for this definition include our respect for and acknowledgement of the Canadian context of care and the Canadian health system. When we report the study findings, we will discuss the differences between the patient definition that we are using and others (e.g. INVOLVE definition) and how our approach affected this scoping review’s approach and results. *Healthcare providers* include licensed health professionals ‘whose practice is based on direct observation and treatment of a patient’ [22]. Health system *decision-makers* are individuals (e.g. members of government, legislature, board directors, healthcare managers, administrators, leaders) who have the authority to set policy regarding the improvement and reform of health care and its delivery. According to CIHR, a *researcher* has to have formal training in research, be employed by an institution and be involved in the intellectual content of the research [23].

##### Concept

Each publication has to include both concepts and/or sub-concepts of *patient-oriented research* or any other similar term (e.g. patient-reported outcomes, patient-centred care) and *competency* or any component of competencies (i.e. *knowledge, skills, attitudes*) that refer to the four stakeholder groups’ competencies in POR. For example, there is a significant body of literature on research competencies for various professionals, but not specific to patient-oriented research. While there is mention of knowledge pertaining to the health system and health research as required competencies, emphasis has also been placed on communication skills and collaboration [10, 24].

##### Context

Boote and colleagues described [25] and defined three levels of public involvement in research: ‘(1) consultation (where researchers seek the views of the public on key aspects of the research); (2) collaboration (an on-going partnership between researchers and the public throughout the research process); (3) “publicly led” (where the public designs and undertakes the research and where researchers are only invited to participate at the invitation of the public)’ [26].

Using Boote’s levels, the context of the proposed scoping review is focusing on the second level: collaboration with the public in governance, priority setting, conducting research and/or knowledge translation (KT) [27] in all settings (e.g. community, acute care, long-term care) without restrictions to the type of setting of the primary publications. The International Association of Public Participation (IAP2) is the preeminent organization of professionals in the field of public participation, who provide the foundational elements (e.g. core values, beliefs, principles) and encourage and support individuals and organizations to adopt and incorporate them into their public-decision processes. IAP2 also advocates for and provides technical assistance to improve public participation, promotes the use of research findings to support educational and advocacy goals; and promotes, improves, advances and extends the practice of public participation in relation to individuals, governments, institutions and other entities that affect the public interest around the world. The public participation goals are to *inform*, *consult*, *involve*, *collaborate*, and *empower* the public. As an advocate, educator and professional development body, IAP2 focuses on developing a broad range of member benefits, services, and information gathering by conducting professional development activities to serve members’ learning needs, promote practical tools and best practices for developing effective public participation processes. The Canadian branch of the International Association of Public Participation, IAP2 Canada (http://iap2canada.ca/page-1020549), is a national organization committed to growing the practice of public administration through engagement, communication and dialogue. IAP2 Canada also guides the BC SUPPORT Unit’s work at the ‘collaborate’ level. The BC SUPPORT Unit, part of Canada’s SPOR led by the CIHR, supports this scoping review for developing and implementing public participation processes to help inform better decisions that reflect the interests and concerns of potentially affected people and organizations.

##### Study design

We will include all study designs including any methodology and type of qualitative, quantitative and mixed methods. We also will include the following types of publication: academic journal papers, grey literature and brief reports focusing on the health sector. Any theoretical frameworks reported in the primary studies will be recorded.

##### Outcomes

The primary endpoints of our interest include core POR competencies for each stakeholder group to engage in POR that are important for decision-making/policy and practice such as *required* knowledge, skills, and competencies. Secondary outcomes include core POR competencies *relevant to* education and future research such as knowledge about research in general and POR in particular; skills for understanding and conducting research; and attitudes towards research and POR. Competencies will be extracted as reported in the included publications. However, if interesting POR competencies are implicitly described, we will extract all relevant composites as reported in individual publications and combine/synthesize them into an umbrella outcome/competency. For example, if elements of efficient communication (e.g. being polite, listening carefully) are described as important components to collaborate with research team members, we may synthesize those components into a broader concept (e.g. communication skills). We will exclude publications that do not report specific or relevant outcomes/POR competencies.

##### Language

Searches will be limited to publications written in English and French. Although none of the research team members is fluent in French, we are interested in identifying relevant literature that might be translated.

##### Timing

Studies will be selected for inclusion from 1990 to 2017. This date is early enough to catch the vast majority of the literature. Restrictions according to status of publication (e.g. in review, accepted, in press) or specific types of records (e.g. commentaries, letters, editorials) will not be applied. Other relevant POR articles will be held in a separate folder to be reviewed as background documents to support the analysis and synthesis of our study.

### Screening and identifying the literature

**Fig. 1:**
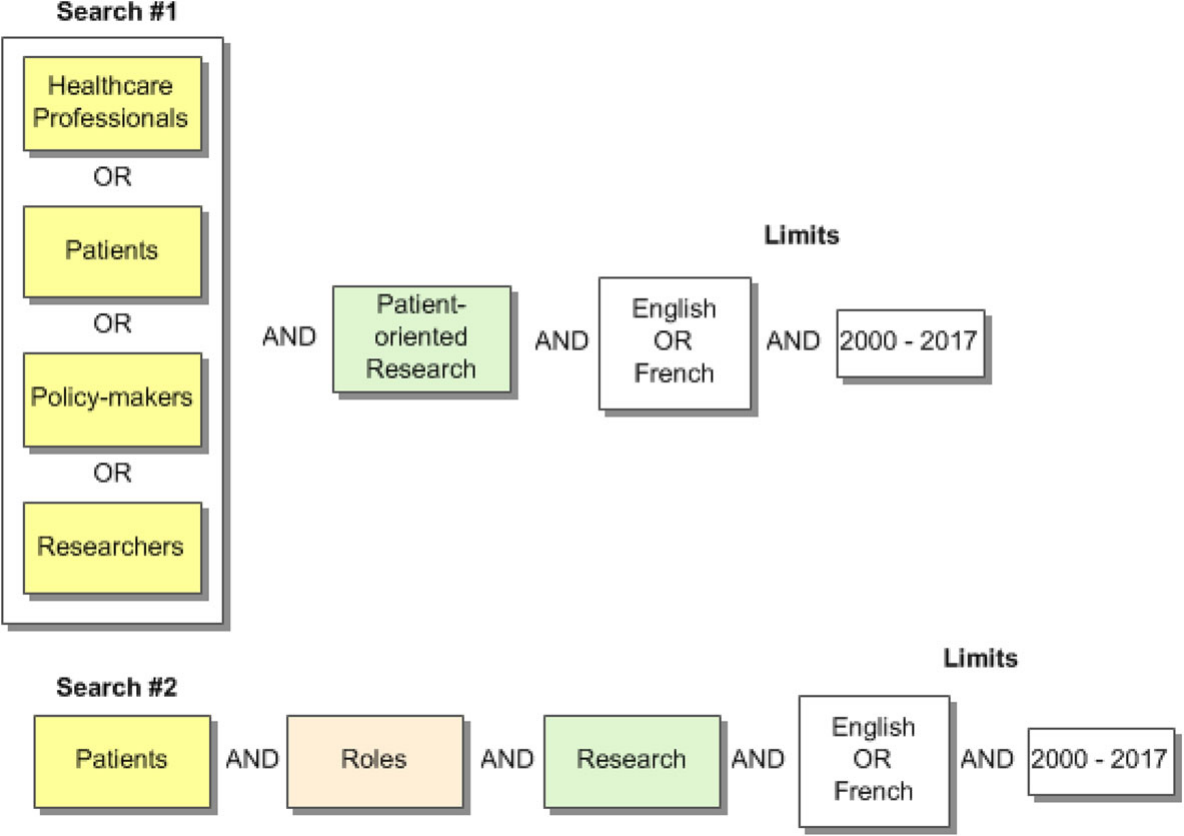
Preliminary search strategy

For the purposes of this scoping review, we will develop a search strategy and refine its parameters in consultation with a research librarian (member of our research team). Then, we will systematically search the academic (peer-reviewed) and grey literature to identify relevant publications. Initial searches indicate that there is very little research that directly focuses on core competencies for patient-oriented research for the four stakeholder groups. This is further complicated by the varying definitions, descriptive terms, and/or elements that are relevant or used to designate patient-oriented research. It is anticipated that competencies will be highlighted within descriptions, evaluations and critiques of patient-oriented research projects. Consequently, several approaches to searching the published literature will be undertaken in order to capture as much of the literature as possible. Searches to date have produced a number of relevant papers. The bibliographic records from these papers have been examined and the subject headings (e.g. MeSH) and keywords from the titles and abstracts of their records have helped in the construction of the initial search concepts. Search one will include the concepts: stakeholders (patients *OR* healthcare professionals/providers *OR* researchers *OR* decision/policy-makers) *AND* patient-oriented research. The second search will include the concepts: patients *AND* roles *AND* research (Fig. 1—preliminary search). The results from these searches will be reviewed and papers found to meet the inclusion criteria will be examined for subject headings and keywords. New searches will be developed to ensure all aspects of the research question have been covered. To ensure high sensitivity in our searches, subject headings and keywords will be included for each concept (see Additional file 3—preliminary list of terms).

While the topic is specifically about health research, healthcare professions span several disciplines such as nursing, medicine, rehabilitation and pharmaceutical sciences, social work, sociology and psychology. Therefore, searches will be undertaken in the databases relevant to these disciplines and available through the University of British Columbia library (see Additional file 4). Key journals will be hand searched to reduce the risk of missed articles (see Additional file 5). References from all papers meeting the inclusion criteria will be reviewed for additional papers. Published papers meeting the inclusion criteria will be searched in the Web of Science, ScienceDirect and Google Scholar for citing papers. The curricula vitae (CVs) of pertinent authors will also be reviewed.

Grey literature searches will be undertaken through several approaches. Internet searches will focus on general searches, patient-oriented research organizations, patient organizations, government websites, training programmes and conferences. Conference abstracts, proceedings and theses will also be searched through appropriate databases. A list of relevant contacts will be developed and then contacted for suggested resources (see Additional file 6). Searching is an iterative process and will continue for both the published and grey literature until sufficient literature has been captured to address the research question. Detailed records of the search process will be recorded to ensure replication. For data management, search results will be imported into a citation management programme (e.g. Mendeley, Zotero) and duplicates removed. References will be exported to at least two reviewers for independent review.

### Selecting relevant publications

All empirical and theoretical/conceptual peer-reviewed publications in the health sector as well as documents from the grey literature that examine POR competencies for the four stakeholder groups will be considered for inclusion. All reports on the same study will be considered, if the relevant outcomes of interest are different; otherwise, only one report will be included in the review. However, duplicate, overlapping, or companion studies (i.e. multiple reports of a single study) may come to light only during the data extraction stage. ‘In this iterative process, retrieved search results will be reviewed for inclusion or exclusion according to the predetermined inclusion/exclusion (eligibility) criteria that were previously described. Prior to commencing the screening and review process, we will train new members of our review team (e.g. graduate students) not familiar with the scoping review process, the citation management programme, and/or the content area. As part of the training process, (a) two reviewers will independently screen a random sample of 5% of the included citations; (b) a calibration exercise will be conducted to ensure reliability in correctly selecting articles for inclusion; and (c) an inter-rater agreement will be calculated. If a low agreement is observed between the reviewers (e.g. a kappa statistic less than 50%), pilot testing will continue until at least 85% agreement is reached. Then, at least two investigators (including doctoral students) will independently screen the titles and abstracts of all search results, based on the pre-defined eligibility criteria. Publications identified as potentially relevant to this review will be retrieved in full text and independently reviewed again against the same eligibility criteria by two reviewers. Disagreements regarding a publication inclusion will be resolved through discussion between the two reviewers or with a third reviewer. We will record the reasons for excluding publications.

### Extracting the data

We will classify the publications into empirical and theoretical peer-reviewed papers (including reports and reviews), and in the grey literature using spreadsheets. Our research team will use a revised data extraction instrument that has been developed for the needs of a previous study [28] using standard formats (see Additional file 7). Charting will be an iterative process at the beginning of the data extraction stage. Data will be entered into Microsoft Excel spreadsheets in tabular format. Prior to commencing the full data extraction, four teams of reviewers will independently extract data from a sample of eligible publications (e.g. 5% of the included citations) to determine the consistency, accuracy and completeness of their approach with the purpose of the review; and to refine the form for capturing all the details of quantitative and qualitative study designs. Data extraction will be carried out in duplicate by independent reviewers to reduce bias and errors in the data extraction process. Data to be extracted include publication information (i.e. study unique identification number, first author’s name, year of publication, publication journal, country, language); study design (i.e. population/sample, stakeholder group, methodology and methods, knowledge syntheses, brief reviews, grey literature, theoretical framework); context (i.e. level of involvement in research, settings, funding sources); concepts (i.e. competencies such knowledge, skills, attitudes on communication, collaboration, and so on and POR or similar concepts such as patient-reported outcomes, patient-centred care); POR competencies (i.e. knowledge, skills, attitudes), and study results/findings per each stakeholder group (i.e. primary relevant to decision-making/policy and practice and secondary relevant to education and future research). In this stage, any duplicate, overlapping, or companion studies may come to light. If so, only one report will be included in the review except if each report refers to different relevant outcomes of interest. In data extraction stage, reviewers will resolve disagreements by discussion. One of two arbitrators (AM or NF) will adjudicate unresolved disagreements. We will contact study authors to resolve any uncertainties via email (i.e. up to three emails 3 weeks apart).

### Collating, summarizing and reporting the results

#### Collating and summarizing findings

We will summarize theoretical and empirical peer-reviewed publications as well as the grey literature in a traditional integrative review [29–31]. We will identify commonalities and differences in constructs/concepts used across studies, map them and collate the data extracted from empirical studies. Then, we will summarize publications and their characteristics in a table (e.g. frequency and type of publications, variables used and defined, study design, measured outcomes, use of theoretical framework) that will constitute our map of the literature. Then, we will combine the findings from both types of the literature (i.e. academic and grey) accordingly using narrative and descriptive summaries as well as an interpretive synthesis.

#### Reporting the results

We will report the findings of the review using tables describing the characteristics of each included publication. In additional tables, we will classify the included publications according to their main characteristics such as participants, study setting, study design, theoretical frameworks used, POR competencies determined in eligible publications, and other findings. We will also draw a conceptual diagram (mind mapping) that will include all identified core POR competencies to illustrate the relationships among the components of the various publications. The mind mapping will be helpful in designing future systematic reviews focused on specific POR competencies that may need enhancement for all four stakeholder groups. Peer-reviewed (academic) and grey literature publications will be described separately, but both will be synthesized and used to identify the core POR competencies. Disagreements will be resolved first by discussion and then by consulting a third reviewer for arbitration.

### Integrated knowledge translation plan

Our research team consists of researchers, knowledge users, healthcare providers, and a librarian; we are in the process of developing a multidisciplinary group of Advisors. The research team and its advisors will regularly collaborate using technology (i.e. WebEx). The discussions, decisions and all relevant documents will be stored on a Web 2.0 website using infrastructure provided by the BC SUPPORT Unit.

Our scheduled KT plan consists of two parts:

1. Advisors—The ‘linkage and exchange model’ [32] informed our establishment of a group of Advisors to ensure that a broad range of international participants (e.g. patients, knowledge users, decision-makers, researchers, healthcare professionals) will contribute to the research process and deliverables of the study. The research team will engage regularly with the Advisors via newsletters and technology to solicit their input on a regular basis. We expect our multidisciplinary Advisors to provide feedback on the research process and deliverables of the scoping review; their expertise and/or insights they might have on the needs of the four stakeholder groups regarding POR competencies; and strategic advice on the interpretation of the study findings and appropriate dissemination approaches to local, national, and international interested individuals and organizations.
2. Dissemination of the findings—Drawing from the Knowledge-to-Action (KTA) framework [33] and the steps of a Planned Action Model [34], we will integrate and use the Dissemination Planning Tool [35] to create a dissemination plan beyond traditional academic methods. For example, opinion leaders can offer an innovative approach to sharing knowledge that has the potential for greater effectiveness than passive approaches (e.g. conference presentations). Our constructed dissemination plan will be used at the end of the scoping review, when the findings are known based on the needs and interests of our intended users/audiences. We will also use the Dissemination Planning Tool [35] with our Advisors to ‘plant the seeds of interest’ [35]. Specifically, we will develop an interactive KT plan by (a) describing key messages emerging from this review; (b) determining the target audiences for each message; (c) identifying the best messenger for each message and audience; (d) involving the Advisors in the development of the dissemination plan and process of spreading the messages arising from the scoping review; and (e) using diverse approaches to disseminate the study findings.

## Discussion

Previous reviews on PPI [36–38] or ‘patient-reported outcome measures’ focused on the impact and economic cost of PPI, the value that lay-volunteers bring to research, evidence-based framework for patient and service user engagement (PSUE) respectively. Our scoping review focuses on POR competencies for four discrete stakeholders/collaborators (i.e. patients, researchers, healthcare providers, and decision-makers). Therefore, the proposed scoping review contributes uniquely to the knowledge of patient-oriented research movement in several ways. In particular,

1. Currently, there is not a comprehensive description of POR competencies for the four stakeholder groups, who are the team members in POR. Our work aims to develop the much needed core competencies across multiple and distinct research team members in POR.
2. This scoping review will map the literature, identify research gaps where primary studies are lacking and needed, and where systematic reviews are required. We anticipate results overlapping POR competencies among the four stakeholder groups, but these findings will lead to subsequent systematic reviews. For example, a future systematic review may focus on unique POR competencies for patients and another may focus on unique POR competencies for researchers.
3. This knowledge synthesis study has the potential to directly influence research funders such as CIHR in developing resources that can be used to increase awareness of POR for addressing complex evidence and/or to hone POR competencies for these four stakeholder groups (i.e. patients, healthcare providers, decision-makers, researchers).
4. Our work will be targeted across a broad scope of health disciplines due to the importance of the findings that can directly inform research, practice and policy-making decisions within these disciplines. Results from this work will be the starting point on how to prepare POR teams and engage relevant stakeholders in clarifying and fulfilling the proposed research agenda.

### Strengths and limitations

This review’s main strength is the use of rigorous and robust methods to select, analyze and synthesize the available literature. Nonetheless, we anticipate several limitations. The first limitation is language due to the inclusion of publications written only in the English and French languages. The second limitation arises from the first review of titles and abstracts, which may not discuss competencies as competencies may not be the key topic of the paper, yet the paper may include competencies and provide valuable information on the topic; this may result in high level of precision, but other eligible publications may be missed. The third limitation is relevant to the research on core competencies for POR that is still in its infancy; so, we will not be able to capture the depth of information that we would expect from a topic that has a significant amount of research. Finally, since the process of scoping reviews does not include a critical appraisal of the included publications, our study findings may lack confidence and validity.

### Implications and recommendations

This scoping review will significantly contribute to the POR initiatives by articulating core POR competencies of each individual stakeholder group, which will describe. These emerging competencies provide a means by which individuals from each stakeholder group can assess their ‘readiness’ to engage in POR and develop their own learning plans to gain competencies they do not hold. The implementation of the POR competencies in practice may positively influence outcomes of the POR initiative. We will also provide recommendations for future research on explicitly identified POR competencies for each of the four stakeholder groups and may be able to determine educational strategies for capacity building within these stakeholder groups. Finally, the findings of this scoping review may guide us in future systematic reviews of the literature on specific POR competencies where sufficient primary studies exist; and inform policy-making process and content.

## Additional files

Additional file 1: Definitions of the Primary PORCH Concepts. A list of the primary PORCH concepts are described/defined for the reader convenience. (PDF 43 kb)

Additional file 2: PRISMA-P checklist. It describes the Preferred Reporting Items for Systematic review and Meta-Analysis Protocols (PRISMA-P) 2015 checklist, which includes recommended items to address in a systematic (scoping) review protocol. (PDF 343 kb)

Additional file 3: Literature Search Strategy. Examples of the literature search strategies are described to be used with electronic databases and grey literature including search terms. (PDF 269 kb)

Additional file 4: Academic Databases. A list of the relevant and available academic databases is described that will be included and used during the literature searches phase of the proposed scoping review. (PDF 207 kb)

Additional file 5: Hand Search Journals. A list of key journals (in alphabetical order) is described that will be hand searched during the literature searches phase of this scoping review. (PDF 98 kb)

Additional file 6: Grey Literature Databases. A list of the relevant and available grey literature databases is described that will be used during the literature searches phase of the scoping review. (PDF 261 kb)

Additional file 7: Data Extraction Form. The data that we will extract from each eligible publication for this scoping review is described in details. (PDF 226 kb)

## Abbreviations

BC SUPPORT Unit: BC Support for People and Patient-Oriented Research and Trials Unit
CIHR: Canadian Institutes of Health Research
CV: Curriculum vitae
IAP2 Canada: The Canadian branch of the International Association of Public Participation
IAP2: International Association of Public Participation
INVOLVE: United Kingdom’s initiative for conducting health research, where ‘patients’ are involved as much and as meaningfully as possible (http://www.invo.org.uk/)
KT: Knowledge translation
KTA: Knowledge-to-Action
PCORI: Patient-Centred Outcomes Research Institute
PE: Patient Engagement
POR: Patient-Oriented Research
PORCH: Patient-Oriented Research Competencies in Health
PRISMA-P: Preferred reporting items for systematic review and meta-analysis protocols
PROSPERO: International Prospective Register of Systematic Reviews (http://www.crd.york.ac.uk/prospero)
SPOR: Strategy for Patient-Oriented Research
