# Supplementary material for: Patient-Oriented Research Competencies in Health (PORCH) for patients, healthcare providers, decision-makers and researchers: protocol of a scoping review
Source: Syst Rev. 2018 Jul 19;7:101. doi: 10.1186/s13643-018-0762-1 (PMC6053801; doi:10.1186/s13643-018-0762-1)
Supplement: Supplementary file 1 — Definitions of the Primary PORCH Concepts. A list of the primary PORCH concepts are described/defined for the reader convenience. (PDF 43 kb) [file 13643_2018_762_MOESM1_ESM.pdf]

Additional File 1 – Definitions

Stakeholders
Any individual, group of individuals, organization or political entity with an interest or stake in the outcome of a decision. Available from: http://iap2canada.ca/Resources/Documents/0702-Foundations-Brochure-MW-FINAL.pdf
In our study, we include patients, healthcare providers, decision-makers (policy-makers), and researchers.

1. **Patient**
"An overarching term inclusive of individuals with personal experience of a health issue and informal caregivers, including family and friends."
Available from: http://www.cihr-irsc.gc.ca/e/48413.html (SPOR).
We interpret this definition broadly to also include those individuals, communities or populations, who have a condition or context of living that makes them vulnerable to health issues (though they may not carry a 'diagnosis' or awareness of a condition) or who are experiencing a normal, healthy life transition that influences health though it may not be understood literally to mean a '*health*' issue or condition (e.g., pregnancy).

2. **Healthcare practitioner/provider**
United States
"Health care provider" is defined as a doctor of medicine or osteopathy, podiatrist, dentist, chiropractor, clinical psychologist, optometrist, nurse practitioner, nurse-midwife, or a clinical social worker who is authorized to practice by the State and performing within the scope of their practice as defined by State law, or a Christian Science practitioner.
Available from: http://hr.berkeley.edu/node/3777
We note that this definition is a legal defintion of those who are generally considered primary care providers (with the exception of the social worker). The National Library of Medicine (Medline Plus) expands this listing by naming healthcare providers who are primary care providers, nursing care providers (licensed nurses and advanced practice nruses) and speciality providers (such as pharmacists).
Available from: https://medlineplus.gov/ency/article/001933.htm

Canada
In the Canada Health Act, a healthcare practitioner means a person lawfully entitled under the law of a province to provide health services in the place in which the services are provided by that person. Available from: http://laws-lois.justice.gc.ca/eng/acts/c-6/page-1.html. Canada's Institute for Health Information (CIHI) includes regulated health professionals as healthcare providers (https://secure.cihi.ca/free_products/hctenglish.pdf) such as registered nurses, physicians and those in other health professions (e.g., pharmacists, chiropractors, dentists).

Australia

A person whose primary employment role is to diagnose physical and mental illnesses, disorders and injuries and prescribe medications and treatments that promote or restore good health. Available from: http://www.aihw.gov.au/medical-practitioner-related-definitions
The Australian Health Practitioner Regulation Agency regulates the practice of many health professionals including physicians, nurses, midwives, occupational, therapists, physical therapists, doctors of oriental medicine and those who are aboriginal health practitioners
Available from: http://www.ahpra.gov.au/Registration/Registers-of-Practitioners/Professions-and-Divisions.aspx

<u>Clinician</u>
"A health professional whose practice is based on direct observation and treatment of a patient, as distinguished from other types of health workers, such as laboratory technicians and those employed in research." (Mosby's Medical Dictionary, 9th edition. © 2009, Elsevier, p. 384). Clinician researchers from all healthcare professions (e.g. nursing, medicine, pharmacy) as well as non-clinicians who conduct applied health research (e.g. clinical epidemiologists, biostatisticians, health economists). Patient-oriented research can be undertaken in all settings including the community (including primary and long-term care) and hospital settings.
Available from: www.nshrf.ca/initiatives/initiatives/strategy-patient-oriented-research-spor

3. **Decision maker**
An individual or professional or community group who makes decisions about, or influences, health policies or practices. Decision-makers can be practitioners, educators, healthcare administrators, elected officials (Exception: Federal elected officials), and individuals within the media, health charities, patient user groups or the private sector. They can work at the local community, municipal, provincial or national level. Decision-makers are those individuals who are likely to be able to make use of the results of the research.
Available from: http://www.cihr-irsc.gc.ca/e/34190.html

4. **Researcher**
<u>Research associate</u>
A researcher who:
- has completed formal training in research in a discipline relevant to health research, usually a Masters or PhD;
- is employed by an institution;
- works under the supervision of a Principal Applicant, i.e., they are not independent researchers;
- may contribute substantially to the intellectual content of the research;
- may contribute to, but not ultimately responsible for, the supervision of staff, including other research associates and trainees, at the discretion of the Principal Applicant.

Available from: http://www.cihr-irsc.gc.ca/e/34190.html

<u>Researcher (independent)</u>
An individual who:
- is autonomous regarding their research activities; and

- has an academic or research appointment which:
  - must commence by the effective date of funding; and
  - allows the individual to pursue the proposed research project, to engage in independent research activities for the entire duration of the funding, to supervise trainees, and to publish the research results; and
  - obliges the individual to conform to institutional regulations concerning the conduct of research, the supervision of trainees, and the employment conditions of staff paid with CIHR funding.

Available from: http://www.cihr-irsc.gc.ca/e/34190.html

Patient-oriented research (POR)
Patient-oriented research refers to a continuum of research that engages patients as partners, focusses on patient-identified priorities and improves patient outcomes. This research, conducted by multidisciplinary teams in partnership with relevant stakeholders, aims to apply the knowledge generated to improve healthcare systems and practices.
Available from: www.cihr-irsc.gc.ca/e/48413.html

Public involvement in research
As research being carried out '*with*' or '*by*' members of the public rather than '*to*', '*about*' or '*for*' them. This includes, for example, working with research funders to prioritise research, offering advice as members of a project steering group, commenting on and developing research materials, undertaking interviews with research participants.
Available from: www.invo.org.uk/find-out-more/what-is-public-involvement-in-research-2/

Public involvement in research is conceptualised as, "doing research 'with' or 'by' the public, rather than 'to', 'about' or 'for' the public". Three main levels of public involvement in research have been differentiated: (1) consultation (where researchers seek the views of the public on key aspects of the research); (2) collaboration (an on-going partnership between researchers and the public throughout the research process); (3) 'publicly led'; where the public designs and undertakes the research and where researchers are only invited to participate at the invitation of the public (Boote, Baird, & Sutton, 2011, p. 107).

Public participation
Any process that involves the public in problem-solving or decision-making and that uses public input to make better decisions. Available from:
http://iap2canada.ca/Resources/Documents/0702-Foundations-Brochure-MW-FINAL.pdf

Research

1. **Research (biomedical)**

Research with the goal of understanding normal and abnormal human functioning, at the molecular, cellular, organ system and whole body levels, including development of tools and techniques to be applied for this purpose; developing new therapies or devices that improve

health or the quality of life of individuals, up to the point where they are tested on human subjects. Studies on human subjects that do not have a diagnostic or therapeutic orientation. Available from: http://www.cihr-irsc.gc.ca/e/34190.html

2. **Research (clinical)**
Research with the goal of improving the diagnosis, and treatment (including rehabilitation and palliation), of disease and injury; and improving the health and quality of life of individuals as they pass through normal life stages. Research on, or for the treatment of, patients. A CIHR theme. Available from: http://www.cihr-irsc.gc.ca/e/34190.html

3. **Research (health services)**
Research with the goal of improving the efficiency and effectiveness of health professionals and the healthcare system, through changes to practice and policy. Health services research is a multidisciplinary field of scientific investigation that studies how social factors, financing systems, organizational structures and processes, health technologies, and personal behaviours affect access to healthcare, the quality and cost of healthcare, and, ultimately, Canadians' health and well-being. A CIHR theme.
Available from: http://www.cihr-irsc.gc.ca/e/34190.html

4. **Research (social, cultural, environmental and population health)**
Research with the goal of improving the health of the Canadian population, or of defined sub-populations, through a better understanding of the ways in which social, cultural, environmental, occupational and economic factors determine health status. A CIHR theme. Available from: http://www.cihr-irsc.gc.ca/e/34190.html

References

Boote, J., Baird, W., & Sutton, A. (2011). Public involvement in the systematic review process in health and social care: A narrative review of case examples. *Health Policy, 102*(2), 105-116. doi:10.1016/j.healthpol.2011.05.002
