# Supplementary material for: Patient-Oriented Research Competencies in Health (PORCH) for patients, healthcare providers, decision-makers and researchers: protocol of a scoping review
Source: Syst Rev. 2018 Jul 19;7:101. doi: 10.1186/s13643-018-0762-1 (PMC6053801; doi:10.1186/s13643-018-0762-1)
Supplement: Supplementary file 3 — Literature Search Strategy. Examples of the literature search strategies are described to be used with electronic databases and grey literature including search terms. (PDF 269 kb) [file 13643_2018_762_MOESM3_ESM.pdf]

Additional File 3 – Possible Search Terms

Patients

- patient, patient's, patients, patients'
- consumer, consumer’s, consumers, consumers’
- public, publics, public’s, publics
- citizen, citizens, citizen’s, citizens’
- stakeholder, stakeholders, stakeholder’s, stakeholders’
- caregiver, caregiver’s, caregivers, caregivers’
- carer, carers, carer’s, carers’
- community
- client
- user
- service user
- lay
- parents
- guardians
- disabled people
- organizations (represent those using services)
- patient advocates
- patient representatives

Clinician

- Physician
- Nurse
- Social Worker
- Allied Health professional
- Physiotherapist
- Occupational therapist

Decision Maker

- decision making
- health care management
- health care planning
- health care policy
- health care quality
- participatory management
- policy maker
- policy making
- public policy

Role

- Engage engages engaged engaging engagement
- Deliberate deliberates deliberated deliberating deliberation
- Participate participates participated participating participation
- Involve involves involved involving involvement
- Collaborate collaborates collaborated collaborating collaboration
- Input
- eliciting
- Partner partners partnered partnering partnership
- Consult consults consulted consulting consultation
- Empower empowers empowered empowering empowerment
- Dialogue dialogues
- Perspective
- Inform
- Shared decision making
- Patient centeredness
- Patient activation

Patient oriented research

Patient oriented research
Patient-outcome research
Patient, person, family, consumer centred care
Patient, person, family, consumer centred medicine
Personalized care
Practiced based research
Clinical research
Translational research
Community participation research

Research (Social, biomedical, medical, health, animal)

- Health services research (HSR)
- health systems research
- health policy and systems research (HPSR)
- medical or biomedical research
- basic science
- bench research
- bench science
- applied research
- pre-clinical research
- clinical research
- basic medical research
- translational research
- Consultation (seeking the view of the public on components of research)

- Collaboration (partnership between researchers & public during research)
- Publicly led (public designs and leads research – researchers invited by public)
- “study design” “trial design” “research design”
- Prioritize prioritizes Priority priorities prioritization priority-setting “priority setting”
- “resource allocation” “allocation of resources”
- Priority setting
- Policy, policy-making
- “topic selection” “selecting topics”
- agenda
- Translate translating Translation
- Disseminate disseminating Dissemination
- Systematic reviews
- Guidelines
- Health technology assessments
- Clinical trials
- Reviews
- Observational studies
- Protocols
- Question development
- Research outcomes
- Implementation plan
- Structures
- Mechanisms
- Evaluation
- Input into organisational governance (organisation wide committee member)
- Fundraising
- Disseminate research information
- Individual research project committee member
- Identify research needs
- Prioritise research
- Input into acceptability of proposed research and likelihood of participation
- Recruit participants
- Other (ethics committee member)
- Assist the development of research funding applications
- Assist the development of research tools e.g. participant surveys or information sheets
- Gather/facilitate research data collection
- Member of research grant review panel
- Contribute to the formulation of research policy such as funding guidelines
- Other (felt laboratory research was not applicable)
- Other (planning to in future)
- Other (provide community talks)
- generating topics, topic generation,

- prioritiz*,
- selecting topics, topic selection, voice
- "Public involvement in the systematic review process is defined as the involvement of the public in one or more of the following review activities:
    - Topic identification, prioritization and commissioning
    - Refinement of review question
    - Development of review protocol (including
    - search strategy and inclusion/exclusion criteria)
    - Literature search
    - Appraisal of the literature, including assessment of study relevance, data extraction and evidence synthesis
    - Initial drafting of findings
    - Interpretation of findings
    - Final report writing
    - advisory board, advisory committee, citizen board, citizen council, citizen jury, focus group, graphic elicitation,
    - grounded theory, ethnography, health experience research, mixed methods research, interview, participatory action research, participatory health research, observation, phenomenology
    - photovoice, photo elicitation
    - patient board, patient council, patient panel, people board, people council, people panel, public board, public council , public panel,
    - questionnaire, social science methods, survey,
    - verbal elicitation,
    - visual elicitation

French

- Consommateurs, patients, publique
- choisir, choix, consultation, consulter, sélection, sélectionner, participation, participer
- la décision médicale partagée, recherche clinique orientée patient, soins orientée patient, sujets de recherche
