# Supplementary material for: Patient-Oriented Research Competencies in Health (PORCH) for patients, healthcare providers, decision-makers and researchers: protocol of a scoping review
Source: Syst Rev. 2018 Jul 19;7:101. doi: 10.1186/s13643-018-0762-1 (PMC6053801; doi:10.1186/s13643-018-0762-1)
Supplement: Supplementary file 4 — Academic Databases. A list of the relevant and available academic databases is described that will be included and used during the literature searches phase of the proposed scoping review. (PDF 207 kb) [file 13643_2018_762_MOESM4_ESM.pdf]

Additional File 4 – Academic Databases

Health Sciences

- MEDLINE (Ovid)
- Embase (Ovid)
- Cochrane Database of Systematic Reviews (Ovid)
- Cochrane Central Register of Controlled Trials (Ovid)
- CINAHL (EBSCO)
- LILACS
- Native Health Database
- Arctic Health Publications
- Health Reference Center
- Health Technology Assessment
- Elsevier ScienceDirect
- TRIP

Social Sciences

- Studies on Women & Gender Abstracts
- Ageline
- Social Work Abstracts
- PsycINFO
- Sociological Abstracts

General

- Web of Science Core Collection
- Academic Search Complete

Theses

- ProQuest Dissertations & Theses Global
- Theses Portal Canada
- British Library EThOS
- National Library of Australia's Trove service

Conference Proceedings

- Web of Science
- Embase (Ovid)
- WorldCat (PapersFirst, ProceedingsFirst)
