# Supplementary material for: Patient-Oriented Research Competencies in Health (PORCH) for patients, healthcare providers, decision-makers and researchers: protocol of a scoping review
Source: Syst Rev. 2018 Jul 19;7:101. doi: 10.1186/s13643-018-0762-1 (PMC6053801; doi:10.1186/s13643-018-0762-1)
Supplement: Supplementary file 5 — Hand Search Journals. A list of key journals (in alphabetical order) is described that will be hand searched during the literature searches phase of this scoping review. (PDF 98 kb) [file 13643_2018_762_MOESM5_ESM.pdf]

Additional File 5 – Hand Search Journals

- Health Services Research
- Health Research Policy and Systems
- Health Policy
- Lancet
- The Patient
- Health Expectations
- Value in health
- Medical Anthropology Quarterly
- Social Science and Medicine
- Culture Medicine and Psychiatry
- Anthropology and Medicine
- Sociology of Health and Illness
