# Supplementary material for: Patient-Oriented Research Competencies in Health (PORCH) for patients, healthcare providers, decision-makers and researchers: protocol of a scoping review
Source: Syst Rev. 2018 Jul 19;7:101. doi: 10.1186/s13643-018-0762-1 (PMC6053801; doi:10.1186/s13643-018-0762-1)
Supplement: Supplementary file 6 — Grey Literature Databases. A list of the relevant and available grey literature databases is described that will be used during the literature searches phase of the scoping review. (PDF 261 kb) [file 13643_2018_762_MOESM6_ESM.pdf]

Additional File 6 – Grey Literature Databases

1. General

- SIGLE
- Grey Literature Report (The New York Academy of Medicine)
- Des Libris
- GreyNet
- Réseau de recherche en santé des populations du Québec
- The Fraser Institute
- PHRED Public Health Research, Education & Development
- Canadian Women's Health Network
- World Health Organization
- OECD iLibrary
- Grey Matters
- Open DOAR
- OAIster
- The Joanna Briggs Institute
- Virtual Health Library
- RIAN (Pathways to Irish Research)
- Lenus (The Irish Health Repository)
- NARCIS - National Academic Research and Collaborations Information System (Netherlands)
- Lenus (The Irish Health Repository)
- INAHTA (International Network of Agencies for Health Technology Assessment)
- Centre for Reviews and Dissemination (UK)
- OpenAIRE
- OpenGrey
- Public health Grey Literature Sources (from OPHLA)

2. Conferences

- Summer Institute 2017

http://www.cvent.com/events/summer-institute-2017-working-together-spotlight-on-patient-oriented-research/custom-37-2d7ea023cd2b493bb95d866dbefc9acd.aspx

- POR Training Program Session 4: Innovative Methods for Patient-Oriented Res

https://www.eventbrite.ca/e/por-training-program-session-4-innovative-methods-for-patient-oriented-research-part-1-identifying-tickets-30329570562?aff=erelexpmlt

- Nova Scotia LGBTQ Health Research Conference June 2916

https://www.dal.ca/diff/gahps/research-projects/improvinglgbtqhealth.htm

- Challenges: the Patients' perspective; IRDiRC meeting, Dublin 2013

3. Training

- http://www.europeanlung.org/assets/files/publications/ubiobookletpip.pdf
- https://www.mcmasterhealthforum.org/citizens/masterclass-patient-oriented-research

- https://www.mailman.columbia.edu/become-student/degrees/masters-programs/master-science/patient-oriented-research
- http://hqca.ca/news/2016/09/event-patient-oriented-research-training-program/
- http://www.aihealthsolutions.ca/initiatives-partnerships/spor/career-development-in-methods-health-services-research-platform/patient-oriented-research-training-program/

4. Organizations

Canadian Consumer Health Associations

- Allergy & Asthma Information Association
- Allergy & Asthma Information Association (ON)
- Alzheimer Society (PEI)
- Alzheimer Society of Canada (NF & LBR)
- Amyotrophic Lateral Sclerosis (ALS) Society
- Anaemia Institute
- Arthritis Society
- Arthritis Society (AB & NWT)
- Arthritis Society (BC & Yukon)
- Arthritis Society (PEI)
- Asthma Society of Canada
- BC Lung Association
- BC Persons with AIDS Society
- Canadian Arthritis Network
- Canadian Arthritis Patient Alliance
- Canadian Association for Independent Living Centres
- Canadian Breast Cancer Network
- Canadian Cancer Society
- Canadian Cancer Society—AB/NWT
- Canadian Cancer Society—NB
- Canadian Cancer Society—NS
- Canadian Cystic Fibrosis Foundation
- Canadian Diabetes Association—ON
- Canadian Diabetes Association (SK)
- Canadian Haemophilia Society
- Canadian Liver Foundation
- Canadian Liver Foundation—BC
- Canadian Liver Foundation—NB
- Canadian Liver Foundation—NS
- Canadian Mental Health Association (MB)
- Canadian Network for Asthma Care
- Canadian Prostate Cancer Network
- Canadian Treatment Action Council (CTAC)

- BC persons with AIDS
- CARP, Canada's Association for the 50 Plus
- Epilepsy Ontario
- Haemophilia Ontario
- Haemophilia Saskatchewan
- Heart & Stroke Foundation
- Heart & Stroke Foundation (PQ)
- Heart & Stroke Foundation (ON)
- Hepatitis C United Resource Exchange (Hep Cure)
- Hepatitis C Resource Centre (MB)
- Kidney Foundation of Canada
- Lupus Canada
- Multiple Sclerosis Society of Canada
- Osteoporosis Society of Canada
- Spina Bifida & Hydrocephalus Association
- Titz n Glitz Breast Cancer

Note: Above list is from (J. Pivik et al. / Health Policy 69 (2004) 253–268 257)

- Health Policy Citzen Council (Toronto)
- CADTH
- Canadian Institutes of Health Research (CIHR)
- Canadian Institute for Health Information (CIHI)
- Centre for health Economics and Policay Analysis (CHEHPA)
- Health Canada
- Health quality council of Ontario
- Institute for Clinical and Evaluative Sciences (ICES)
- Institute of Research and Public Policy

<u>United States (US)</u>

- Patient-Centered Outcomes Research Institute (PCORI)
- Kaiser Permanente
- National Health and Medical Research Council (NHMRC)
- National Institutes of Health
- Agency for Healthcare Research & Quality
- Centre for health Services and Policy Research
- National Guidelines Clearinghouse
- IOM Institute of Medicine
- NIHR National Institute for Health Research

<u>United Kingdon (UK)</u>

- INVOLVE
- Wellcome Trust
- National Health Service (NHS)

- National Institute for Health Research (NIHR)
- Mental Health Research Network (MHRN)
- Mental Health Foundation
- www.peopleinresearch.org/
- www.ukctg.nihr.ac.uk/pages/signup-page
- www.nihr.ac.uk/patients-and-public/
- www.hra.nhs.uk/resources/public-involvement-research/
- Medical Research Council
- Centre for Health Information Quality (UK)
- Consumers in NHS Research Support Unit
- United Patients' Organisations of the Chronically Ill (WOCZ)
- Research Institute for Consumer Affairs (RICA) (1960's ??)
- National Consumer Council
- Patients Association
- National Institute for Health and Clinical Excellence (NICE)
- James Lind Alliance
- SCH Scottish Health Services
- Medical Research Council
- Health Technology Assessment (HTA) Programme - NIHR

<u>Europe</u>

- Picker Institute Europe
- European patients forum
- *European* Medicines Agency (EMA) (Eligible patients and consumers organisations
- Cochrane Consumer network
- Julius Center for Patient-Oriented Research, Utrecht University Medical School, Utrecht, the Netherlands
- "Centre de sociologie de l'innovation" (Ecole des Mines, Paris, France)
- European Organisation for Rare Diseases (EURORDIS)
- The European Patient Voice
- AQUMED German Agency for Quality in Medicine (Ärztliches Zentrum für Qualität in der Medizin)
- ECRIN EFGCP European Clinical Research Infrastructure Network European Forum for Good Clinical Practice
- EFSA European Food Safety Authority
- EGAN European Genetic Alliances Network
- EMA European Medicines Agency
- ENPCR European Network of Patients Partnering in Clinical Research
- EPF European Patients' Forum
- EUNETHTA European Network for Health Technology Assessment
- EUPATI European Patients Academy on Therapeutic Innovation

- EURORDIS IAPO European Organization for Rare Diseases International Alliance of Patients' Organizations
- VSOP Dutch Genetic Alliance

<u>Australia</u>

- ACSQHC Australian Commission on Safety and Quality in Health Care
- National Health and Medical Research Council (NHMRC)
- Cancer Voices Australia
- The Consumers' Health Forum of Australia

<u>International Organizations</u>

- Cochrane Collaboration
- International Alliance of Patients' Organizations
- ICPHR International Collaboration on Participatory Health Research
- ICPHR International Collaboration on Participatory Health Research
