# Supplementary material for: Patient-Oriented Research Competencies in Health (PORCH) for patients, healthcare providers, decision-makers and researchers: protocol of a scoping review
Source: Syst Rev. 2018 Jul 19;7:101. doi: 10.1186/s13643-018-0762-1 (PMC6053801; doi:10.1186/s13643-018-0762-1)
Supplement: Supplementary file 7 — Data Extraction Form. The data that we will extract from each eligible publication for this scoping review is described in details. (PDF 226 kb) [file 13643_2018_762_MOESM7_ESM.pdf]

Additional File 7 – Data Extraction Form

Reviewer:	
Date:	
**Publication Information**	
Study unique ID:	
1st Author's Name:	
Publication Year:	Mainly between 1990-2017
Journal:	
Country:	
Language written:	English, French, Other
**Study design**	
Population/Sample:	Demographics, Not applicable
Stakeholder group:	Patient, healthcare provider, decision-maker, researcher
Methodology & methods:	Qualitative (QL), quantitative (QN), mixed methods (MM), not applicable (e.g., grey literature)
Knowledge synthesis:	Systematic review, scoping review, etc.
Brief review:	Conference proceedings, etc.
Grey literature:	Editorial, commentary, etc.
Theoretical framework:	Yes (specify), None
**Context**	
Level of involvement in research:	Consultation; Collaboration (in governance, priority setting, conducting research, conducting KT activities); Publicly led POR
Setting:	Community, acute care, long-term care, etc.
Funding sources:	
**Concepts**	
Competencies:	Knowledge, skills, attitudes (e.g., on communication, collaboration)
POR or similar:	Patient-reported outcomes, patient-centered care, etc.
**POR competencies**	
Knowledge (specify):	•<br>•<br>•
Skills (specify):	•<br>•<br>•
Attitudes (specify):	•<br>•<br>•
Other (specify):	•<br>•<br>•

Study Results/Findings	
Main/Primary findings per each stakeholder group (specify):	Relevant to Decision-making/Policy:<br>•<br>•<br>•<br>Relevant to Practice<br>•<br>•<br>•
Secondary findings per stakeholder group (specify):	Relevant to Education<br>•<br>•<br>•<br>Relevant to Research<br>•<br>•<br>•
Other findings (specify):	•<br>•<br>•

Conclusion	
Include:	
Exclude (provide rationale):	
Additional Comments:	
